# Immediate and Midterm Cardiac Remodeling After Surgical Pulmonary Valve Replacement in Adults With Repaired Tetralogy of Fallot: A Prospective Cardiovascular Magnetic Resonance and Clinical Study

**DOI:** 10.1161/CIRCULATIONAHA.117.027402

**Published:** 2017-10-30

**Authors:** Ee Ling Heng, Michael A. Gatzoulis, Anselm Uebing, Babulal Sethia, Hideki Uemura, Gillian C. Smith, Gerhard-Paul Diller, Karen P. McCarthy, Siew Yen Ho, Wei Li, Piers Wright, Veronica Spadotto, Philip J Kilner, Paul Oldershaw, Dudley J. Pennell, Darryl F. Shore, Sonya V. Babu-Narayan

**Affiliations:** From Adult Congenital Heart Disease Centre, (E.L.H., M.A.G., A.U.., B.S., H.U., W.L., V.S., P.O., D.F.S., S.V.B.-N.), Cardiac Morphology Unit (K.P.M., S.Y.H.), and Non-Invasive Cardiology Department (P.W.), Royal Brompton Hospital, London, United Kingdom; National Institute for Health Research Cardiovascular Biomedical Research Unit, Royal Brompton & Harefield NHS Foundation Trust and Imperial College London, United Kingdom (E.L.H., M.A.G., G.C.S., P.J.K., D.J.P., D.F.S., S.V.B.-N.); Division of Adult Congenital and Valvular Heart Disease, Department of Cardiovascular Medicine, University Hospital of Münster, Germany (G.-P.D.); and Department of Thoracic and Cardiovascular Sciences, University of Padua, Italy (V.S.).

**Keywords:** magnetic resonance imaging, pulmonary valve, tetralogy of Fallot, ventricular remodeling

## Abstract

Pulmonary valve replacement (PVR) in patients with repaired tetralogy of Fallot provides symptomatic benefit and right ventricular (RV) volume reduction. However, data on the rate of ventricular structural and functional adaptation are scarce. We aimed to assess immediate and midterm post-PVR changes and predictors of reverse remoeling.

**Methods:**

Fifty-seven patients with repaired tetralogy of Fallot (age ≥16 y; mean age, 35.8±10.1 y; 38 male) undergoing PVR were prospectively recruited for cardiovascular magnetic resonance performed before PVR (pPVR), immediately after PVR (median, 6 d), and midterm after PVR (mPVR; median, 3 y).

**Results:**

There were immediate and midterm reductions in indexed RV end-diastolic volumes and RV end-systolic volumes (RVESVi) (indexed RV end-diastolic volume pPVR versus immediately after PVR versus mPVR, 156.1±41.9 versus 104.9±28.4 versus 104.2±34.4 mL/m^2^; RVESVi pPVR versus immediately after PVR versus mPVR, 74.9±26.2 versus 57.4±22.7 versus 50.5±21.7 mL/m^2^; *P*<0.01). Normal postoperative diastolic and systolic RV volumes (the primary end point) achieved in 70% of patients were predicted by a preoperative indexed RV end-diastolic volume ≤158 mL/m^2^ and RVESVi ≤82 mL/m^2^. RVESVi showed a progressive decrease from baseline to immediate to midterm follow-up, indicating ongoing intrinsic RV functional improvement after PVR. Left ventricular ejection fraction improved (pPVR versus mPVR, 59.4±7.6% versus 61.9±6.8%; *P*<0.01), and right atrial reverse remodeling occurred (pPVR versus mPVR, 15.2±3.4 versus 13.8±3.6 cm^2^/m^2^; *P*<0.01). Larger preoperative RV outflow tract scar was associated with a smaller improvement in post-PVR RV/left ventricular ejection fraction. RV ejection fraction and peak oxygen uptake predicted mortality (*P*=0.03) over a median of 9.5 years of follow-up.

**Conclusions:**

Significant right heart structural reverse remodeling takes place immediately after PVR, followed by a continuing process of further biological remodeling manifested by further reduction in RVESVi. PVR before RVESVi reaches 82 mL/m^2^ confers optimal chances of normalization of RV function.

## Abstract 2

Supplemental Digital Content is available in the text.

**Clinical Perspective**

**What Is New?**

- Cardiac remodeling has been regarded as a gradual process after surgical pulmonary valve replacement (PVR) in patients with repaired tetralogy of Fallot. We demonstrated immediate structural right heart reverse remodeling after surgery.
- Progressive reduction in indexed right ventricular end-systolic volumes was demonstrated for the first time, likely reflecting improved intrinsic right ventricular contractility after PVR.
- Improved left-sided heart hemodynamics and right atrial remodeling were evident after surgical PVR.
- Preoperative right ventricular ejection fraction and peak oxygen uptake on cardiopulmonary exercise testing predicted all-cause mortality during postoperative follow-up.

**What Are the Clinical Implications?**

- The immediate postoperative changes in right ventricular volumes and function after surgical PVR provide mechanistic insight into the early reverse remodeling process.
- There is potential for ongoing reverse right ventricular remodeling once the volume load of pulmonary regurgitation is removed.
- Intervening before the indexed right ventricular end-systolic volume reaches 82 mL/m^2^ guides timing of surgical PVR to optimize chances of right ventricular normalization after intervention.

Relief of right ventricular (RV) outflow tract (RVOT) obstruction at the time of surgical repair of tetralogy of Fallot frequently leads to pulmonary regurgitation (PR) with its lifelong adverse consequences. Although PR is generally well tolerated in the short term, with time, it leads to progressive RV dilatation, clinical decline (exercise intolerance, arrhythmia, and heart failure), and an increased risk of sudden cardiac death.^1–5^

Pulmonary valve replacement (PVR) is undertaken to mitigate these late effects with reported symptomatic benefits, in conjunction with improved RV volumes and left ventricular (LV) function.^3,6,7^ Although the overall hemodynamic benefits of PVR are evident with broad consensus for surgery before clinical deterioration or overt symptoms develop, uncertainties remain about the optimal timing of PVR, as well as the timing and nature of reverse ventricular remodeling.

We aimed in this study to assess prospectively immediate and midterm cardiac remodeling after surgical PVR using cardiovascular magnetic resonance (CMR).

## Methods

### Patient Population and Study Design

Patients with repaired tetralogy of Fallot (rTOF) scheduled for elective PVR who were ≥16 years of age with no contraindications to CMR and estimated glomerular filtration rate ≥30 mL/min under the care of the Adult Congenital Heart Disease Center, Royal Brompton Hospital, UK, were approached for study inclusion between January 2005 and December 2009. Individualized recommendations for PVR were made after multidisciplinary reviews. During this period, our group started applying published preoperative RV threshold volume cutoffs of indexed RV end-diastolic volume (RVEDVi) ≥150 mL/m^2^ and RV end-systolic volume (RVESVi) ≥80 mL/m^2^ among other clinical parameters (onset of arrhythmia, impaired exercise capacity, or other symptoms) for referral to PVR. The study complies with the Declaration of Helsinki and has local ethics committee approval. All participants provided study-specific written informed consent.

CMR (1.5 T; Siemens) was performed at 3 time points: preoperatively, immediately postoperatively before hospital discharge, and midterm postoperatively (minimum, 12 months) after surgery, the last per routine clinical follow-up. Baseline demographics of study participants and clinical observations were obtained preoperatively and at midterm follow-up. Echocardiography was performed by a single operator for assessment of RV restrictive physiology with laminar anterograde flow in the pulmonary artery in late diastole present throughout the respiratory cycle.^8^ Cardiopulmonary exercise testing for measurements, including peak oxygen uptake and ratio of minute ventilation to carbon dioxide production was carried out with the modified Bruce protocol to symptom limitation. Tests were excluded from analysis if the respiratory quotient value was <1. Clinical follow-up data were collated for the occurrence of death and cardiac events (death, sustained ventricular tachycardia, documented sustained atrial arrhythmias, and new-onset heart failure requiring hospitalization or diuretics).

Reverse RV remodeling was defined as the reduction in noninvasively measured CMR RV volumes after PVR, widely regarded as a beneficial positive outcome of surgery. The primary end point was normalization of RV volumes at the midterm follow-up as defined by RVEDVi ≤108 mL/m^2^ and RVESVi ≤47 mL/m^2^ (normal RV volume range established in our institution with the same method).^9^ Secondary end points were percentage change in RVEDVi and RVESVi and myocardial fibrosis burden assessed by late gadolinium enhancement (LGE) CMR as a predictor of postsurgical remodeling.

### CMR Protocol

All participants underwent standardized full CMR assessment, including short-axis steady-state free-precession cine stack from the atrioventricular ring to the apex. Biventricular volumes, mass, and function were derived with semiautomated software (CMRtools, Cardiovascular Imaging Solutions). A single core laboratory observer completed all volumetric measurements on anonymized scans. Twelve study CMR scans were randomly selected and remeasured by the same experienced observer (with a minimum 6-month interval) and a second blinded experienced observer for ventricular volume reproducibility assessment. LV papillary muscles and major trabeculations of the RV were carefully excluded from the blood pool and considered part of the myocardial mass. Maximal right atrial (RA) area index was recorded from a 4-chamber view. RA dilatation is defined as indexed RA area ≥15 cm^2^/m^2^.^10^ Akinesia was defined as noncontractile myocardium that did not thicken in systole. Indexed RA area >16 cm^2^/m^2^ and RVOT akinetic length >30 mm were also recorded given their predictive value.^11^ Pulmonary regurgitant fraction was calculated from phase-velocity mapping in a plane transecting the main pulmonary trunk. Corrected right ventricular ejection fraction (RVEF) was calculated by dividing the net pulmonary flow (pulmonary forward flow minus the regurgitant flow) by the RVEDV.^12^ LGE imaging was acquired by a single operator at preoperative and midterm postoperative time points as previously described.^13^ Nonspecific LGE at RV/LV insertion points (present in 98%) was excluded.^13,14^ LGE was present if bright signal within myocardium was visualized with good-quality images in the same region on phase swap or cross-cuts. The maximum linear extent of RVOT LGE was measured. The LGE findings of 12 scans were rescored by investigators blinded to previous analysis (with a minimum 6-month interval).^13^

### Quantification of RV Resected During RVOT Reconstruction

Study participants donated resected tissue necessarily removed at the time of surgery (typically in the RVOT). The area of resected RVOT tissue was measured against carefully aligned linear scales to include maximum specimen length and height to derive total tissue area before fixation.

### Statistical Analysis

Data normality was assessed by Kolmogorov-Smirnov analysis. Results are reported as mean±SD or median (interquartile range) according to data distribution. Groups were assessed by paired Student *t* tests or the Wilcoxon signed-rank test as appropriate. Categorical variables were compared by χ^2^ and McNemar tests as appropriate. Bonferroni correction was applied when multiple comparisons were undertaken by dividing the original α value of 0.05 by the number of analyses on the dependent variable (k). CMR parameters were grouped to determine k because CMR constituted the main imaging modality in our study for assessing cardiac remodeling after PVR. Other clinical and imaging parameters, including echocardiography, were regarded as a separate group for correction. Correlations were assessed by Pearson and Spearman correlation tests.

In view of the range of time intervals at midterm follow-up, a mixed-effects model was applied to adjust for the effects of time on derived CMR parameters. A repeated-measures mixed-effects linear model was also used to test the association between surgically resected RVOT area and change in RV volumes and function over time. Covariables, including preoperative biventricular volumes and function (indexed end-diastolic volume, indexed end-systolic volume, and ejection fraction), were included as fixed effects, along with time of follow-up as a continuous variable in the analysis, whereas individual subjects were considered random effects. An unstructured covariance structure was used for analysis. This choice was supported by assessment of the model fit based on the Akaike information criterion.

Receiver-operating characteristic curves analysis was performed to assess the cutoff values that predicted normalization of RV volumes after PVR. The optimal cutoff threshold was determined by the Youden index (J), which is represented by maximum sensitivity plus specificity minus 1. The predictive value of preoperative parameters on clinical outcomes was assessed by univariate Cox hazards regression analysis. The proportional hazards assumption was verified for each variable with generalized linear regression analysis, testing for a nonzero slope of the scaled Schoenfeld residuals in addition to visual inspections of the graphs of the regression. The correlations between preoperative clinical parameters (CMR and echocardiographic data) and RV normalization were assessed by univariable binary logistic regression analyses.

For the assessment of intraobserver and interobserver reproducibility, variability was expressed as the coefficient of variation (percent), derived from the within-subject SD divided by the mean multiplied by 100%. The within-subject SD is as follows: √∑ (difference between paired measurements)^2^/2n.^15^ Intraclass correlation coefficients were also assessed on the basis of an absolute agreement, 2-way mixed-effects model. Statistical analysis was performed with SPSS (IBM Statistics V.22) and R (version 3.1.2). A 2-sided value of *P*<0.05 was considered statistically significant.

## Results

### Patient Characteristics

Fifty-seven patients with rTOF were prospectively recruited (age, 35.8±10.1 years; age at repair, 4.0 years [interquartile range, 0.3–36.5 years]; 38 male). Their baseline clinical and surgical characteristics are listed in Table 1.

**Table 1.**
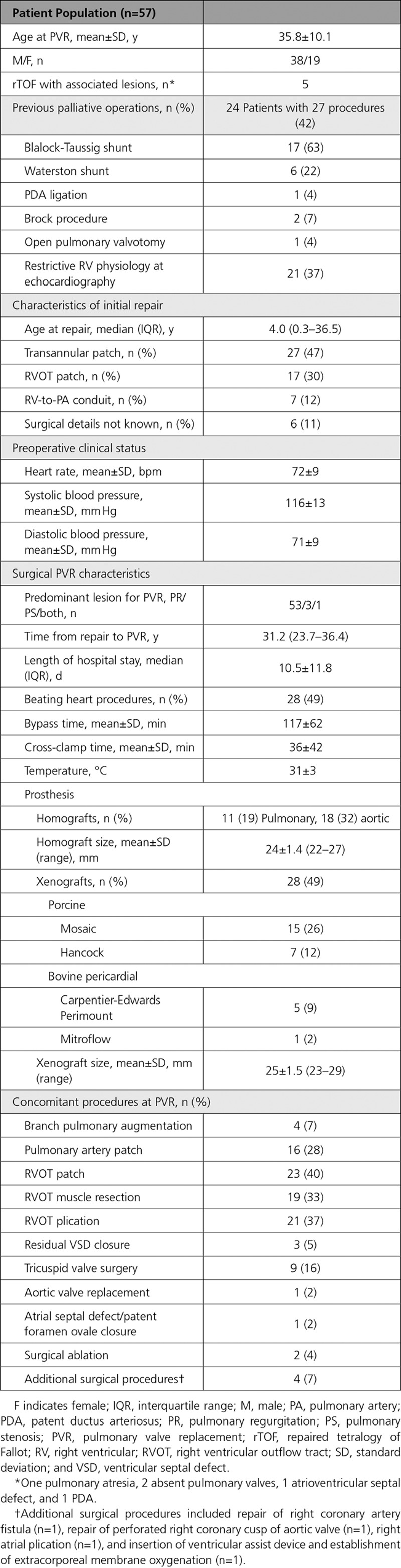
Baseline Characteristics and PVR Surgical Details

### Clinical Data, Exercise Tolerance, QRS Duration, and Cardiothoracic Ratio

There were 2 early postoperative deaths (both from RV failure at day 3) and 1 sudden death at 34 days (Table I in the online-only Data Supplement).

Seventy-four percent of patients were symptomatic at baseline (predominant symptom of dyspnea/fatigue in 56%, documented arrhythmia in 11%, palpitations in 5%, and endocarditis in 2%), but only 2% were symptomatic at midterm follow-up after PVR. Peak and percent predicted oxygen uptake (n=27 paired data with respiratory exchange ratio >1) remained unchanged (pre-PVR versus post-PVR, 23.7±5.8 versus 24.3±7.8 mL·kg^−1^·min^−1^, *P*=0.67; and 73.1±17.9% versus 73.4±16.7%, *P*=0.93), as did ratio of minute ventilation to carbon dioxide production (pre-PVR versus post-PVR, 34.3±13.2 versus 34.0±6.9; *P*=0.92) and ventilatory threshold (pre-PVR versus post-PVR, 16.2±4.6 versus 16.2±4.1 mL·kg^−1^·min^−1^; *P*=0.95). Right bundle-branch block morphology on ECGs with baseline QRS duration of 155±21 milliseconds did not change after PVR (*P*=0.73). Cardiomegaly (cardiothoracic ratio ≥0.50) was noted in 81% (46 patients) preoperatively and reduced significantly after PVR (*P*<0.01;Table 2). Restrictive physiology was documented in 29% of patients before PVR and 15% of patients at midterm follow-up (Table 2).

**Table 2.**
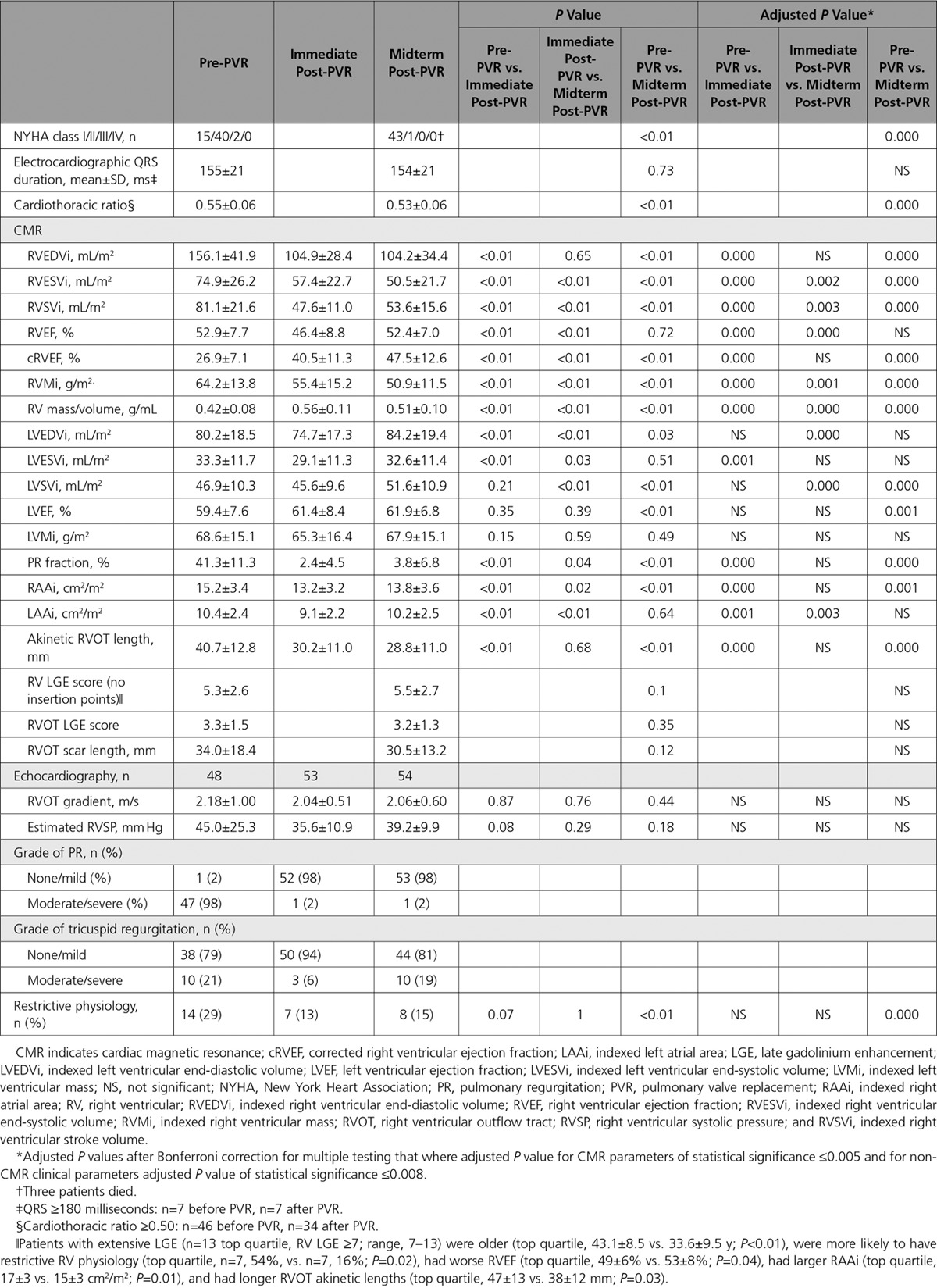
Electrocardiographic, Cardiothoracic Ratio, Echocardiographic, and CMR Data at Baseline and After PVR

Eighteen patients (32%) experienced major adverse clinical events (4 deaths, 5 sustained ventricular tachycardia, 14 new-onset sustained atrial arrhythmias, 1 new-onset heart failure). Preoperative RVEF and peak oxygen uptake on exercise emerged as univariable predictors of death during follow-up, and RVEF predicted a combined end point of death or sustained ventricular tachycardia (*P*=0.01). Table 3 lists the statistically significant predictors of adverse clinical outcomes, and Table II in the online-only Data Supplement provides a comprehensive list of all clinical variables examined. Predictors of atrial arrhythmia were also identified. No statistically significant difference in clinical outcomes during follow-up associated with preoperative RV volumes was found (Table 4).

**Table 3.**
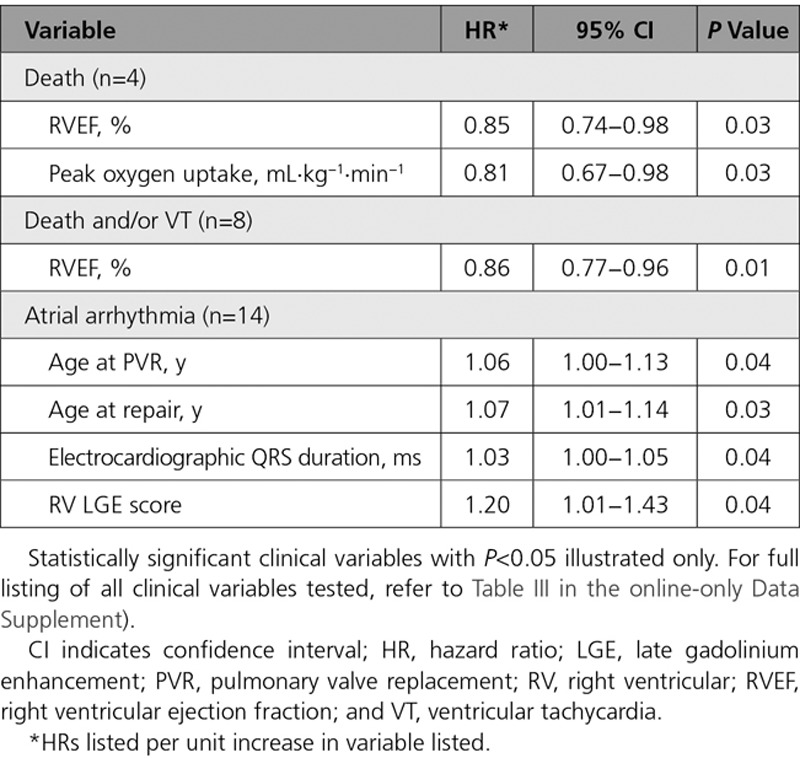
Univariable Cox Regression Analysis for Adverse Clinical Outcomes

**Table 4.**
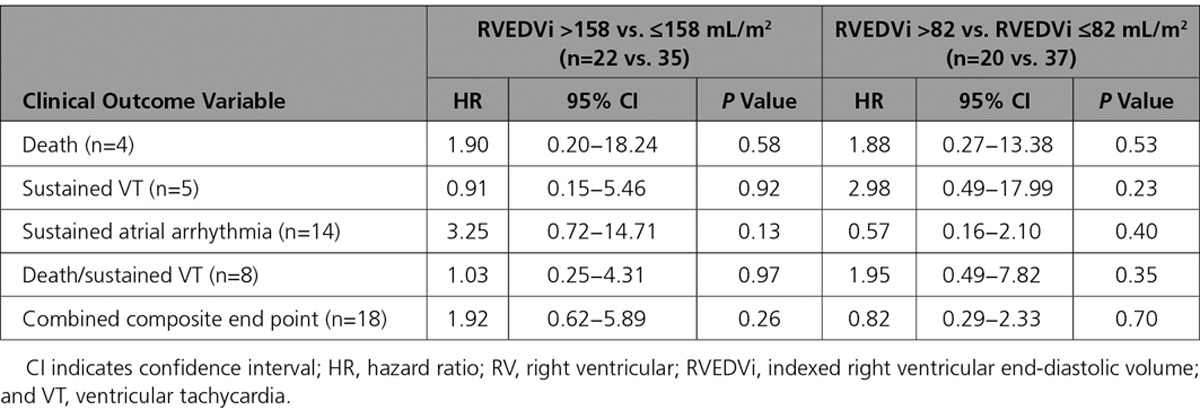
Univariable Cox Regression Analysis of Relationship Between Preoperative RV Volumes and Adverse Clinical Outcomes During Follow-Up

### Cardiovascular Magnetic Resonance

Immediate postoperative CMRs were performed at 6 days (interquartile range, 5–7.5 d; n=46) and midterm CMRs at 3.0 years (interquartile range, 1.0–4.1 y; n=47) after the index PVR. Post-PVR CMRs were not performed because of implantable cardioverter-defibrillators (n=6), death (n=3), and a move overseas (n=1).

### RV and RA Reverse Remodeling

**Figure 1.**
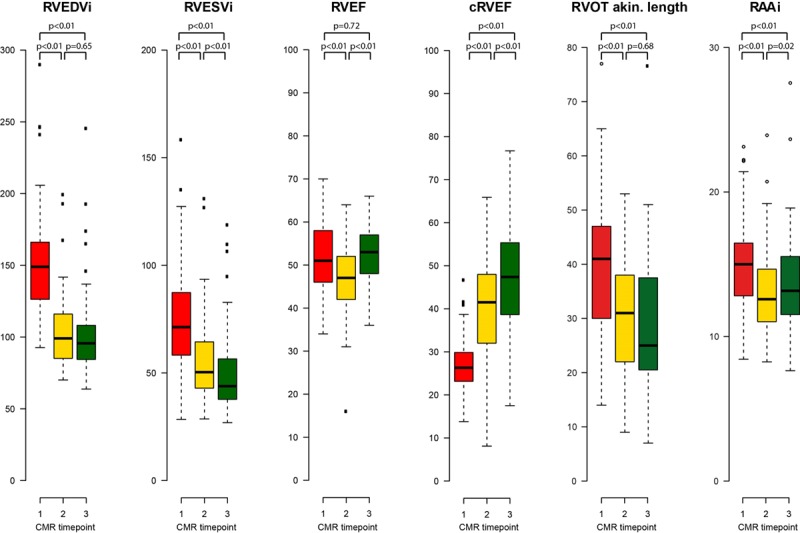
**Right heart cardiovascular magnetic resonance (CMR) parameters.** Study time points: red, before pulmonary valve replacement (PVR); yellow, immediately after PVR within 14 days; green, at midterm follow-up after PVR. cRVEF indicates corrected right ventricular ejection fraction; RAAi, indexed right atrial area; RVEDVi, indexed right ventricular end-diastolic volume; RVEF, right ventricular ejection fraction; RVESVi, indexed right ventricular end-systolic volume; and RVOT akin., linear length of akinetic right ventricular outflow tract.

RV volumetric and RA reverse remodeling data are summarized in Table 2 and Figure 1. In the immediate post-PVR period (median, 6 days), there was a 32% reduction in RVEDVi (*P*<0.01), which was sustained at midterm follow-up. RVESVi promptly decreased by 23% (*P*<0.01) and decreased further by midterm follow-up (*P*<0.01) to 32% lower than the baseline (Table 2 and Figure 1).

**Figure 2.**
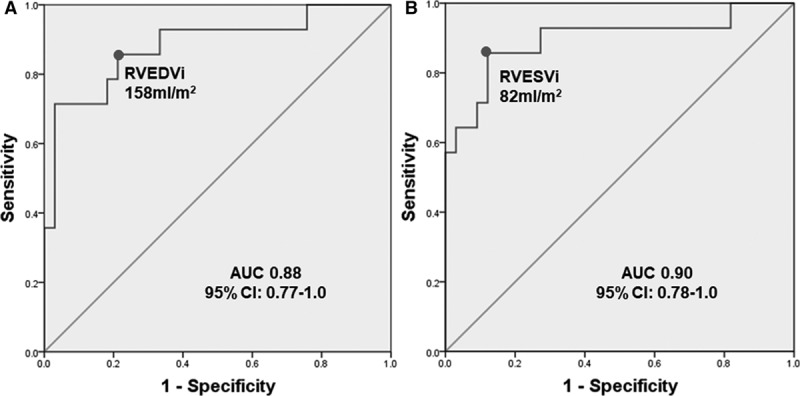
**Receiver-operating characteristic curves illustrating threshold value.** (**A**) Pre–pulmonary valve replacement (PVR) indexed right ventricular end-diastolic volume (RVEDVi) of 158 mL/m^2^ for normalization of RVEDVi and (**B**) pre-PVR indexed right ventricular end-systolic volume (RVESVi) of 82 mL/m^2^ for normalization of RVESVi. The 95% confidence intervals (CIs) for area under the curve (AUC) are given.

Normalization of RV volumes (both RVEDVi ≤108 mL/m^2^ and RVESVi ≤47 mL/m^2^) was achieved in 33 patients (70%) at midterm follow-up. Preoperative RVEDVi and RVESVi values were used to predict RV normalization. The optimal cutoff values were calculated with receiver-operating characteristic curve analysis, for which the highest sensitivity and specificity were achieved. These were RVEDVi of 158 mL/m^2^ (sensitivity, 86%; specificity, 79%) and RVESVi of 82 mL/m^2^ (sensitivity, 86%; specificity, 88%; Figure 2). Preoperative RV volumes and function, biventricular mass, RA size, and peak RVSP on echocardiography influenced RV normalization as shown in Table 5.

**Table 5.**
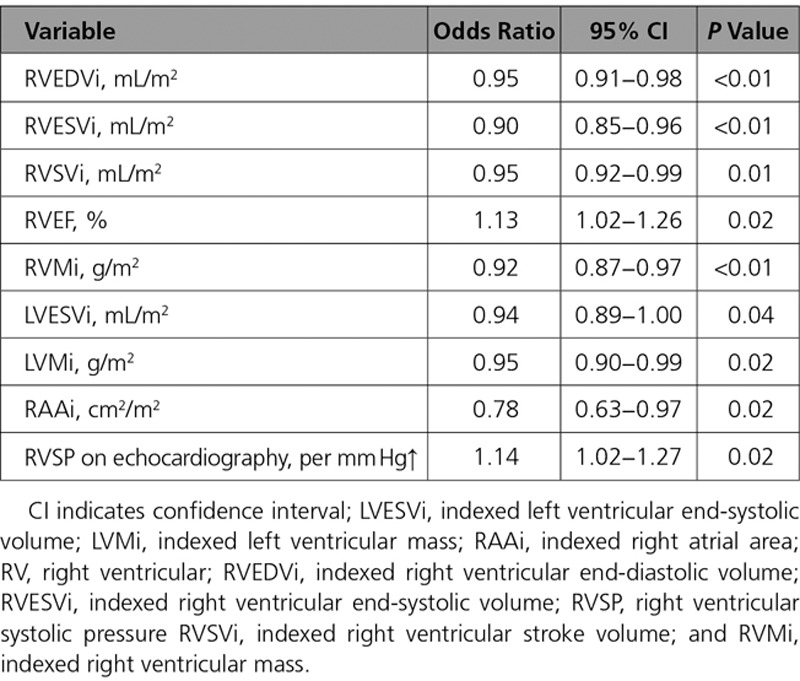
Univariable Logistic Regression Analysis for Predictors of RV Normalization

RVEF decreased immediately by 12% after PVR but returned back to baseline preoperative levels by midterm follow-up. RV volumetric changes were accompanied by a regression of indexed RV mass at both post-PVR time points (*P*<0.01). Indexed RA area decreased after PVR with a corresponding decrease in the prevalence of RA dilatation (*P*<0.01 for both; Table 2 and Figure 1).

### RVOT Akinetic Regions and RV LGE Extent

The area of surgically resected RVOT quantified on linear scales did not correlate with reduction in RV volumes or functional change at midterm follow-up (linear mixed-effects model; *P*>0.05). In contrast, RVOT akinetic length on CMR decreased immediately after PVR and was sustained at midterm after PVR (*P*<0.01; Table 2 and Figure 1). Pre-PVR RVOT akinetic length was modestly associated with change in QRS duration after PVR (*r*=0.44, *P*<0.01).

All patients had RV LGE (median RV score, 5; range, 1–13) at sites of previous surgical intervention (RVOT, ventricular septal defect regions), whereas RV LGE remote from surgical sites was present in 13 patients (23%) only. Four patients had nonapical vent-related LV LGE (median LV LGE score, 9.5; range, 7–20). Total RV LGE scores did not change after PVR (*P*=0.10 and *P*=0.35), nor did linear extent of RVOT scar (*P*=0.12; Table 2). There was an inverse relationship between pre-PVR linear RVOT scar length and change in biventricular EFs (ΔEF=midterm post-PVR EF−pre-PVR EF) with ΔRVEF (*r*=−0.40, *P*<0.01) and Δleft ventricular ejection fraction (LVEF) (*r*=−0.41, *P*<0.01).

### LV Volumetric Data

Although surgical intervention was confined to the right side of the heart, changes in LV volumes and function were evident (Table 2). Indexed left ventricular end-diastolic and end-systolic volumes (LVEDVi and LVESVi) reduced immediately after PVR and increased thereafter, with indexed left ventricular end-diastolic volumes increasing above its preoperative value. Left ventricular ejection fraction increased after PVR above pre-PVR baseline (Table 2). There was no overall change in mean indexed left atrial areas with surgery.

### Variability in CMR Measurements

Intraobserver and interobserver reproducibility of indexed RV measurements was as follows: 0.9% and 3.1%, 2.8% and 7.9%, 3.4% and 8.5%, 2.9% and 7.4%, and 3.2% and 20.8% for EDV, ESV, stroke volume, EF, and mass, respectively. The intraclass correlation coefficients for LGE scores were 0.94 for the RV and 1.0 for the LV, comparable to the high reproducibility previously reported.^13^

## Discussion

Our data demonstrate for the first time that the majority of improvement in RV volumes after surgical PVR, previously shown at ≥6 months after surgery, takes place very early, within days from restoring pulmonary valve competency, and is sustained thereafter. The immediate effect of PVR on RV volumes is related to the immediate reduction in volume overload. Furthermore, RVESVi continues to decrease, suggesting ongoing functional RV recovery and underscoring RVESVi as a marker of myocardial function. Increases in indexed left ventricular end-diastolic volumes and left ventricular ejection fraction at midterm follow-up, although modest, are indicative of a positive ventricular interdependence effect after PVR and improved left heart hemodynamics.

### Evidence of Time-Dependent Reverse RV Systolic Volume Remodeling

Most of the decrease in RVEDVi (32%) took place in the immediate postoperative period compared with a mere further 2% reduction at midterm follow-up. There was also a rapid reduction of RVESVi in the early post-PVR period (24%), but this was followed by a further 9% reduction to midterm. The latter reflects extended recovery of RV function with time. Our data suggest that the changes in RVEDVi observed are likely to reflect acute mechanical remodeling from volume reduction, whereas the later and progressive improvement of RVESVi may represent time-dependent biological remodeling—that is, continued improvement of load-independent RV function. RVESVi has been suggested as a valid estimate of intrinsic, load-independent RV function in rTOF.^16^ Our data support this notion that RVESVi is a measure of RV myocardial function. We suggest that future studies of the timing of PVR should focus on the presence and degree of any continued reduction in RVESVi during serial post-PVR follow-up because changes in RVESVi are independent of ventricular loading conditions.

Postoperative reduction of RV volumes into the normal range—that is, normalization—considered an optimal PVR outcome, took place in the majority of our patients (70%). Our cutoffs for RV volumes that predicted RV volume normalization (RVEDVi ≤158 mL/m^2^, RVESVi ≤82 mL/m^2^) are in close agreement with previously reported preoperative thresholds of RVEDVi of 150 to 163 mL/m^2^ and RVESVi of 80 to 85 mL/m^2^.^17–22^ Higher preoperative RVESVi has been identified as an important predictor of post-PVR RVEF, and it has therefore been suggested that greater emphasis should be placed on RVESVi values in terms of timing of PVR.^19,23^ Indeed, the identified preoperative RVESVi cutoff was equally sensitive but more specific (sensitivity, 86%; specificity, 88%) for normalization of RV volumes compared with the identified RVEDVi cutoff (sensitivity, 86%; specificity, 79%).

When patients with rTOF undergoing PVR for PR were compared, the significant reduction in RVEDVi demonstrated immediately after surgical PVR was sustained to midterm follow-up, similar to changes reported early (<1 mo) and at 1 year after percutaneous pulmonary valve implantation.^24^ However, the early decrease in RVESVi seen in patients with percutaneous pulmonary valve implantation did not show ongoing improvement at 1 year, in contrast to the progressive reduction in RVESVi seen in our surgical PVR cohort.^24^ The changes in RV volumes we found were accompanied by a stepwise reduction in indexed RV mass after PVR. This can be explained by chronic volume loading with PR, resulting in sarcomeric replication in series with chamber enlargement and a proportional increase in wall thickness in response to the increased end-diastolic wall stress. Restoring pulmonary valve competency with PVR initiates regression of ventricular mass as an adaptive response to normalize stress per unit myocardial mass.^25^

### RVOT and PVR in rTOF

We quantified the area of resected RVOT tissue as a measure (albeit coarse) of the extent of RVOT surgical remodeling carried out, as judged appropriate by the operating surgeon. However, tissue area measurements did not appear to influence the extent of RV volumetric or functional reverse remodeling seen after PVR. This is in keeping with the findings of a randomized trial of PVR with and without RV remodeling surgery.^20^ It may also reflect intrinsic measurement bias from 2-dimensional area quantification of irregularly shaped masses of resected tissue or variations in practice among different surgeons.

Postoperative CMRs showed a significant reduction in the length of the RVOT akinetic area. Increased preoperative RVOT akinetic lengths correlated with greater reduction in postoperative QRS duration, presumably as a result of surgical resection of slow-conducting, fibrotic/patch tissue in RVOT akinetic regions.^26^ We previously showed that larger CMR RVOT akinetic length predicts sustained ventricular tachycardia,^11^ whereas in the present study, it predicts postoperative reduction in QRS duration. QRS duration is a recognized sudden death risk factor in rTOF.^4,24,27^ Greater preoperative LGE CMR–defined RVOT scar length was associated with smaller postoperative improvement in both RV and LV ejection fractions, suggesting that more extensive preoperative RV LGE may not be amenable to surgical remodeling and may predict suboptimal PVR outcomes. Reassuringly, worsening of overall RV LGE scores did not occur.

### RA Reverse Remodeling

Indexed RA areas were mildly dilated in our study cohort and decreased after PVR. The fate of the RA during postoperative cardiac remodeling is important because RA enlargement is predictive of atrial tachyarrhythmia, especially in patients with RV restrictive physiology.^11,28^ The positive effect of PVR on RA size merits further work focusing on RA function and its relationship to outcomes after PVR.

### Evidence of LV Reverse Remodeling

Although the focus of PVR outcomes has historically centered on the RV, LV remodeling also occurs. Previous studies have described the association between significant PR and LV systolic dysfunction and the prognostic importance of LV function in rTOF.^2,29^ Although PVR directly exerts right heart alterations, our study, like others, demonstrated increased LV volumes^20,21^ and improved LV function,^18^ similar to findings after percutaneous pulmonary valve implantation.^24^ In addition to any reversed Bernheim effect in which RV geometric and functional adaptations lead to LV dysfunction,^3^ increased LV filling may be achieved as a result of improved efficiency of RV cardiac output when a competent pulmonary valve is implanted. This larger preload translates to an increase in LVEDV and function. This may contribute to patients reporting improved exercise tolerance, although we found no objective improvement of peak oxygen uptake on cardiopulmonary exercise testing in our study. The unchanged peak oxygen uptake was anticipated given that PR was the predominant lesion in our study cohort and that improvements may be the result of recovery kinetics after exercise versus peak oxygen uptake.^30,31^ As we and others have previously reported,^30–32^ however, patients in this study had marked improvement in symptoms at midterm follow-up. Preoperative peak oxygen uptake predicted mortality in our study, in keeping with our previous findings in patients undergoing surgical PVR.^32^

### Limitations

Because the study midterm CMR follow-up took place at the time of routine clinical follow-up, there was variation in the time frame that this occurred. However, we sought to address any confounding through a time-adjusted linear-effects model.

Patients with implanted electronic devices were excluded, and there was necessary attrition of study participants who had pacemakers or implantable cardioverter-defibrillators inserted after study recruitment. This may have introduced bias by excluding patients at the extreme end of the clinical spectrum with significant ventricular dysfunction or other adverse risk markers who required primary or secondary prevention against malignant arrhythmias. Patients who were recruited to the clinical trial were not otherwise different from all patients undergoing surgical PVR in the study period (Table III in the online-only Data Supplement).

### Conclusions

In our single-center prospective study, we have demonstrated pertinent and novel immediate changes after surgical PVR, directly reflecting biventricular and RA remodeling. Normalization of RV volumes, an objective after PVR, was achieved in 70% of study patients and correlated with baseline RVEDVi/RVESVi. Although this early and desirable response to volume offloading was sustained at midterm follow-up, we additionally observed a continuing reduction in RVESVi with a concomitant midterm recovery in RVEF, suggesting time-related biological remodeling of the load-independent RV function after PVR. A preoperative RVESVi cutoff of ≤82 mL/m^2^ was equally sensitive and more specific for normalization of RV volumes compared with our preoperative RVEDVi threshold of ≤158 mL/m^2^, justifying the use of RVESVi for clinical timing of PVR to achieve optimal reverse remodeling.

## Sources of Funding

This work was supported by the British Heart Foundation (FS/13/76/30477 to Dr Heng and FS/11/38/28864 to Dr Babu-Narayan) and the National Institute for Health Research Cardiovascular Biomedical Research Unit of the Royal Brompton & Harefield National Health Service Foundation Trust and Imperial College London.

## Disclosures

The views expressed in this publication are those of the author(s) and not necessarily those of the National Health Service, the National Institute for Health Research, or the Department of Health. Dr Pennell is a consultant to Siemens and a shareholder and director of Cardiovascular Imaging Solutions. The other authors report no conflicts.

## Supplementary Material

## References

[1] Murphy JG, Gersh BJ, Mair DD, Fuster V, McGoon MD, Ilstrup DM, McGoon DC, Kirklin JW, Danielson GK (1993). Long-term outcome in patients undergoing surgical repair of tetralogy of Fallot.. N Engl J Med.

[2] Geva T, Sandweiss BM, Gauvreau K, Lock JE, Powell AJ (2004). Factors associated with impaired clinical status in long-term survivors of tetralogy of Fallot repair evaluated by magnetic resonance imaging.. J Am Coll Cardiol.

[3] Geva T (2011). Repaired tetralogy of Fallot: the roles of cardiovascular magnetic resonance in evaluating pathophysiology and for pulmonary valve replacement decision support.. J Cardiovasc Magn Reson.

[4] Gatzoulis MA, Balaji S, Webber SA, Siu SC, Hokanson JS, Poile C, Rosenthal M, Nakazawa M, Moller JH, Gillette PC, Webb GD, Redington AN (2000). Risk factors for arrhythmia and sudden cardiac death late after repair of tetralogy of Fallot: a multicentre study.. Lancet.

[5] Harrild DM, Berul CI, Cecchin F, Geva T, Gauvreau K, Pigula F, Walsh EP (2009). Pulmonary valve replacement in tetralogy of Fallot: impact on survival and ventricular tachycardia.. Circulation.

[6] Ferraz Cavalcanti PE, Sá MP, Santos CA, Esmeraldo IM, de Escobar RR, de Menezes AM, de Azevedo OM, de Vasconcelos Silva FP, Lins RF, Lima Rde C (2013). Pulmonary valve replacement after operative repair of tetralogy of Fallot: meta-analysis and meta-regression of 3,118 patients from 48 studies.. J Am Coll Cardiol.

[7] Cheung EW, Wong WH, Cheung YF (2010). Meta-analysis of pulmonary valve replacement after operative repair of tetralogy of Fallot.. Am J Cardiol.

[8] Gatzoulis MA, Clark AL, Cullen S, Newman CG, Redington AN (1995). Right ventricular diastolic function 15 to 35 years after repair of tetralogy of Fallot: restrictive physiology predicts superior exercise performance.. Circulation.

[9] Maceira AM, Prasad SK, Khan M, Pennell DJ (2006). Reference right ventricular systolic and diastolic function normalized to age, gender and body surface area from steady-state free precession cardiovascular magnetic resonance.. Eur Heart J.

[10] Maceira AM, Cosín-Sales J, Roughton M, Prasad SK, Pennell DJ (2013). Reference right atrial dimensions and volume estimation by steady state free precession cardiovascular magnetic resonance.. J Cardiovasc Magn Reson.

[11] Bonello B, Kempny A, Uebing A, Li W, Kilner PJ, Diller GP, Pennell DJ, Shore DF, Ernst S, Gatzoulis MA, Babu-Narayan SV (2013). Right atrial area and right ventricular outflow tract akinetic length predict sustained tachyarrhythmia in repaired tetralogy of Fallot.. Int J Cardiol.

[12] Vliegen HW, van Straten A, de Roos A, Roest AA, Schoof PH, Zwinderman AH, Ottenkamp J, van der Wall EE, Hazekamp MG (2002). Magnetic resonance imaging to assess the hemodynamic effects of pulmonary valve replacement in adults late after repair of tetralogy of Fallot.. Circulation.

[13] Babu-Narayan SV, Kilner PJ, Li W, Moon JC, Goktekin O, Davlouros PA, Khan M, Ho SY, Pennell DJ, Gatzoulis MA (2006). Ventricular fibrosis suggested by cardiovascular magnetic resonance in adults with repaired tetralogy of Fallot and its relationship to adverse markers of clinical outcome.. Circulation.

[14] Babu-Narayan SV (2010). The role of late gadolinium enhancement cardiovascular magnetic resonance in the assessment of congenital and acquired heart disease.. Prog Pediatr Cardiol.

[15] Jones RG, Payne RB (1997). Clinical Investigation and Statistics in Laboratory Medicine.

[16] Uebing A, Fischer G, Schlangen J, Apitz C, Steendijk P, Kramer HH (2011). Can we use the end systolic volume index to monitor intrinsic right ventricular function after repair of tetralogy of Fallot?. Int J Cardiol.

[17] Therrien J, Provost Y, Merchant N, Williams W, Colman J, Webb G (2005). Optimal timing for pulmonary valve replacement in adults after tetralogy of Fallot repair.. Am J Cardiol.

[18] Oosterhof T, van Straten A, Vliegen HW, Meijboom FJ, van Dijk AP, Spijkerboer AM, Bouma BJ, Zwinderman AH, Hazekamp MG, de Roos A, Mulder BJ (2007). Preoperative thresholds for pulmonary valve replacement in patients with corrected tetralogy of Fallot using cardiovascular magnetic resonance.. Circulation.

[19] Lee C, Kim YM, Lee CH, Kwak JG, Park CS, Song JY, Shim WS, Choi EY, Lee SY, Baek JS (2012). Outcomes of pulmonary valve replacement in 170 patients with chronic pulmonary regurgitation after relief of right ventricular outflow tract obstruction: implications for optimal timing of pulmonary valve replacement.. J Am Coll Cardiol.

[20] Geva T, Gauvreau K, Powell AJ, Cecchin F, Rhodes J, Geva J, del Nido P (2010). Randomized trial of pulmonary valve replacement with and without right ventricular remodeling surgery.. Circulation.

[21] Frigiola A, Tsang V, Bull C, Coats L, Khambadkone S, Derrick G, Mist B, Walker F, van Doorn C, Bonhoeffer P, Taylor AM (2008). Biventricular response after pulmonary valve replacement for right ventricular outflow tract dysfunction: is age a predictor of outcome?. Circulation.

[22] Bokma JP, Winter MM, Oosterhof T, Vliegen HW, van Dijk AP, Hazekamp MG, Koolbergen DR, Groenink M, Mulder BJ, Bouma BJ (2016). Preoperative thresholds for mid-to-late haemodynamic and clinical outcomes after pulmonary valve replacement in tetralogy of Fallot.. Eur Heart J.

[23] Henkens IR, van Straten A, Schalij MJ, Hazekamp MG, de Roos A, van der Wall EE, Vliegen HW (2007). Predicting outcome of pulmonary valve replacement in adult tetralogy of Fallot patients.. Ann Thorac Surg.

[24] Lurz P, Nordmeyer J, Giardini A, Khambadkone S, Muthurangu V, Schievano S, Thambo JB, Walker F, Cullen S, Derrick G, Taylor AM, Bonhoeffer P (2011). Early versus late functional outcome after successful percutaneous pulmonary valve implantation: are the acute effects of altered right ventricular loading all we can expect?. J Am Coll Cardiol.

[25] Grossman W (1980). Cardiac hypertrophy: useful adaptation or pathologic process?. Am J Med.

[26] Uebing A, Gibson DG, Babu-Narayan SV, Diller GP, Dimopoulos K, Goktekin O, Spence MS, Andersen K, Henein MY, Gatzoulis MA, Li W (2007). Right ventricular mechanics and QRS duration in patients with repaired tetralogy of Fallot: implications of infundibular disease.. Circulation.

[27] Gatzoulis MA, Till JA, Somerville J, Redington AN (1995). Mechanoelectrical interaction in tetralogy of Fallot: QRS prolongation relates to right ventricular size and predicts malignant ventricular arrhythmias and sudden death.. Circulation.

[28] Khairy P, Aboulhosn J, Gurvitz MZ, Opotowsky AR, Mongeon FP, Kay J, Valente AM, Earing MG, Lui G, Gersony DR, Cook S, Ting JG, Nickolaus MJ, Webb G, Landzberg MJ, Broberg CS, Alliance for Adult Research in Congenital Cardiology (AARCC) (2010). Arrhythmia burden in adults with surgically repaired tetralogy of Fallot: a multi-institutional study.. Circulation.

[29] Ghai A, Silversides C, Harris L, Webb GD, Siu SC, Therrien J (2002). Left ventricular dysfunction is a risk factor for sudden cardiac death in adults late after repair of tetralogy of Fallot.. J Am Coll Cardiol.

[30] Coats L, Khambadkone S, Derrick G, Sridharan S, Schievano S, Mist B, Jones R, Deanfield JE, Pellerin D, Bonhoeffer P, Taylor AM (2006). Physiological and clinical consequences of relief of right ventricular outflow tract obstruction late after repair of congenital heart defects.. Circulation.

[31] Lurz P, Riede FT, Taylor AM, Wagner R, Nordmeyer J, Khambadkone S, Kinzel P, Derrick G, Schuler G, Bonhoeffer P, Giardini A, Daehnert I (2014). Impact of percutaneous pulmonary valve implantation for right ventricular outflow tract dysfunction on exercise recovery kinetics.. Int J Cardiol.

[32] Babu-Narayan SV, Diller GP, Gheta RR, Bastin AJ, Karonis T, Li W, Pennell DJ, Uemura H, Sethia B, Gatzoulis MA, Shore DF (2014). Clinical outcomes of surgical pulmonary valve replacement after repair of tetralogy of Fallot and potential prognostic value of preoperative cardiopulmonary exercise testing.. Circulation.

