# Supplementary material for: Immediate and Midterm Cardiac Remodeling After Surgical Pulmonary Valve Replacement in Adults With Repaired Tetralogy of Fallot: A Prospective Cardiovascular Magnetic Resonance and Clinical Study
Source: Circulation. 2017 Oct 30;136(18):1703–13. doi: 10.1161/CIRCULATIONAHA.117.027402 (PMC5662153; doi:10.1161/CIRCULATIONAHA.117.027402)
Supplement: Supplementary file 1 [file cir-136-1703-s001.pdf]

SUPPLEMENTAL MATERIAL

Supplementary Table 1: Baseline CMR and cardiopulmonary exercise test data for deceased study patients

Patient	Age (years)	Gender	***Baseline CMR Data*** RVEDVi (ml/m^2)	RVESVi (ml/m^2)	RVEF (%)	LVEF (%)	PRF (%)	RV LGE score *	RVOT scar length (mm)	***Cardiopulmonary Exercise Test Data*** Peak VO_2 (ml/kg/min)	% predicted peak VO_2	VE/VCO_2 slope	AT (ml/kg/min)
1	43	Female	131.2	71.3	42	50	33.3	6	45	8.0	39	55	7.2
2	24	Male	179.2	78.4	56	60	43.6	4	36	28.1	60	29	16.3
3	36	Female	148.6	84.9	43	66	33.8	6	55	12.5	53	53	8.6

* RV LGE score (no insertion points)

EDVi, indexed end-diastolic volume; EF, ejection fraction; ESVi, indexed end-systolic volume; LGE, late gadolinium enhancement; LV, left ventricle; PRF, pulmonary regurgitant fraction; RV, right ventricle; RVOT, right ventricular outflow tra

Supplementary Table 2: Year on year comparison of study participants with remaining patients with rTOF who underwent PVR 2005-2009 demonstrating lack of recruitment bias

**Patients with rTOF**	**2005**			**2006**			**2007**			**2008**			**2009**		
	n = 19			n = 19			n = 26			n = 25			n = 27		
	Study	***Non-Study***	***p***	***Study***	***Non-Study***	***p***	***Study***	***Non-Study***	***p***	***Study***	***Non-Study***	***p***	***Study***	***Non-Study***	***p***
Age, years	38.3±8.5	34.2±16.6	0.63	32.1±11.5	31.0±10.0	0.85	34.1±7.9	30.3±10.3	0.32	34.3±13.0	27.3±8.8	0.12	38.2±9.9	30.8±9.2	0.06
Gender, male/female	6/8	2/3	1.00	5/9	3/2	0.60	9/1	7/9	0.04	6/3	10/6	1.00	8/3	7/9	0.24
Symptoms (NYHA≥2), n	9	2	1.00	9	2	1.00	8	12	0.56	5	6	1.00	5	4	1.00
QRS duration, ms	149±29	113±21	0.06	160±22	178±2	0.28	148±26	147±32	0.92	160±13	162±25	0.84	165±9	144±32	0.07
<u>*CMR*</u>	n = 14	n=3		n = 14	n = 3		n = 10	n = 12		n = 9	n = 11		n = 11	n =12	
RVEDVi, ml/m^2	161±57	218±158	0.60	157±37	165±71	0.87	163±47	140±65	0.36	153±25	168±61	0.48	148±30	163±28	0.24
RVESVi, ml/m^2	91±38	134±120	0.60	76±19	85±39	0.55	90±30	81±47	0.62	85±17	101±50	0.37	81±25	81±18	0.99
RVSVi, ml/m^2	71±21	84±45	0.67	75±23	79±32	0.79	73±26	58±23	0.17	68±23	67±23	0.98	67±15	81±13	**0.02**
RVEF, %	45±5	42±19	0.84	48±10	48±3	0.99	45±8	43±9	0.64	44±11	41±9	0.52	46±8	50±5	0.1
LVEDVi, ml/m^2	73±16	55±53	0.62	76±22	91±19	0.30	77±21	69±11	0.28	82±19	77±23	0.64	74±21	82±18	0.37
LVESVi, ml/m^2	29±11	20±22	0.31	34±19	34±10	0.94	33±13	30±10	0.55	38±13	34±13	0.46	32±18	31±11	0.91
LVSVi, ml/m^2	45±8	35±31	0.63	43±11	57±10	0.05	44±10	36±12	0.12	45±13	44±14	0.84	44±10	51±10	0.12
LVEF, %	62±7	66±11	0.54	61±8	63±4	0.64	58±8	58±8	0.93	55±12	57±8	0.64	61±11	62±7	0.68
LVMi, g/m^2	63±15	81±47	0.68	60±14	72±26	0.25	78±16	59±16	**0.02**	72±20	60±15	0.15	67±16	58±11	0.14
Pulmonary regurgitant fraction, %	38±9	50±6	0.05	45±10	31±19	0.09	39±7	39±10	0.97	36±9	40±16	0.55	37±17	42±5	0.34

Supplementary Table 3: Univariable Cox regression analysis of clinical variables against clinical outcomes of **(A)** death, **(B)** VT **(C)** death and/or VT **(D)** atrial arrhythmias and **(E)** combined composite endpoint of death, VT and/or atrial arrhythmias.

	(A) Death			*(B) VT*			*(C) Death and/or VT*			*(D) Atrial Arrhythmia*			*(E) Composite Endpoint*		
**Variable**	**HR**	**95% CI**	**p value**	**HR**	**95% CI**	**p value**	**HR**	**95% CI**	**p value**	**HR**	**95% CI**	**p value**	**HR**	**95% CI**	**p value**
Age at PVR, years	1.01	0.92 - 1.12	0.81	1.03	0.95 - 1.13	0.47	1.03	0.96 - 1.10	0.47	1.06	1.00 - 1.13	**0.04**	1.03	0.99 - 1.09	0.18
Age at repair, years	0.95	0.74 - 1.22	0.69	1.03	0.91 - 1.17	0.65	1.01	0.90 - 1.14	0.83	1.07	1.01 - 1.14	**0.03**	1.05	0.99 - 1.12	0.13
ECG QRS duration, ms	1.02	0.97 - 1.07	0.45	1.03	0.99 - 1.07	0.15	1.03	0.99 - 1.06	0.10	1.03	1.00 - 1.05	**0.05**	1.02	1.00 - 1.04	0.11
**CMR**															
RVEDVi, ml/m^2	1.00	0.97 - 1.03	0.89	1.00	0.99 - 1.02	0.68	1.00	0.99 - 1.02	0.78	1.00	0.99 - 1.01	0.77	1.00	0.99 - 1.01	0.74
RVESVi, ml/m^2	1.01	0.98 - 1.05	0.52	1.01	0.99 - 1.04	0.33	1.01	0.99 - 1.04	0.28	1.00	0.98 - 1.02	0.87	1.00	0.98 - 1.02	0.83
RVEF, %	0.85	0.74 - 0.98	**0.03**	0.86	0.73 - 1.01	0.07	0.86	0.77 - 0.96	**0.01**	0.92	0.84 - 1.01	0.09	0.93	0.86 - 1.00	0.06
RVMi, g/m^2	0.96	0.91 - 1.01	0.09	0.97	0.91 - 1.03	0.26	0.99	0.94 - 1.04	0.59	1.00	0.96 - 1.04	0.96	0.98	0.95 - 1.02	0.36
LVEDVi, ml/m^2	0.98	0.92 - 1.04	0.44	1.02	0.97 - 1.06	0.50	1.01	0.97 - 1.04	0.81	1.00	0.97 - 1.03	0.74	1.00	0.97 - 1.02	0.84
LVESVi, ml/m^2	0.96	0.86 - 1.06	0.37	0.99	0.90 - 1.08	0.75	0.98	0.92 - 1.05	0.56	1.01	0.95 - 1.06	0.82	1.00	0.95 - 1.04	0.87
LVEF, %	1.03	0.91 - 1.18	0.68	1.11	0.96 - 1.29	0.16	1.06	0.95 - 1.18	0.29	0.96	0.88 - 1.04	0.30	1.00	0.93 - 1.07	0.89
LVMi, g/m^2	0.97	0.91 - 1.04	0.41	1.02	0.97 - 1.08	0.45	1.01	0.96 - 1.06	0.75	1.00	0.97 - 1.04	0.96	1.00	0.97 - 1.03	0.97
RAAi, cm^2/m^2	1.10	0.83 - 1.45	0.52	1.02	0.81 - 1.29	0.85	1.09	0.90 - 1.31	0.38	1.03	0.89 - 1.18	0.73	1.00	0.88 - 1.14	0.96
LAAi, cm^2/m^2	0.83	0.52 - 1.33	0.45	0.93	0.60 - 1.45	0.75	0.91	0.65 - 1.28	0.60	0.95	0.72 - 1.24	0.68	0.92	0.72 - 1.16	0.47
Akinetic RVOT length, mm	0.95	0.87 - 1.04	0.28	0.99	0.92 - 1.07	0.88	0.98	0.92 - 1.04	0.44	1.03	0.99 - 1.07	0.18	1.01	0.98 - 1.05	0.52
RV LGE score	0.92	0.60 - 1.41	0.70	1.21	0.91 - 1.61	0.18	1.09	0.85 - 1.39	0.51	1.20	1.01 - 1.43	**0.04**	1.15	0.98 - 1.34	0.10
RVOT scar length	1.02	0.96 - 1.08	0.59	1.02	0.96 - 1.08	0.47	1.01	0.97 - 1.06	0.62	1.02	0.98 - 1.05	0.30	1.01	0.98 - 1.04	0.39
**CPEX**															
Peak VO_2, ml/kg/min	0.81	0.67 - 0.98	**0.03**	1.03	0.86 - 1.22	0.78	0.93	0.82 - 1.06	0.30	0.95	0.85 - 1.06	0.36	0.98	0.89 - 1.07	0.58
VE/VCO_2 slope	1.05	1.00 - 1.11	0.08	1.02	0.95 - 1.10	0.55	1.03	0.98 - 1.08	0.19	1.04	1.00 - 1.08	0.09	1.03	0.99 - 1.07	0.10
Ventilatory threshold, ml/kg/min	0.78	0.59 - 1.03	0.08	0.98	0.77 - 1.26	0.88	0.90	0.75 - 1.08	0.27	0.89	0.76 - 1.05	0.16	0.90	0.79 - 1.03	0.14
